# High-Quality Text-to-Speech Implementation via Active Shallow Diffusion Mechanism

**DOI:** 10.3390/s25030833

**Published:** 2025-01-30

**Authors:** Junlin Deng, Ruihan Hou, Yan Deng, Yongqiu Long, Ning Wu

**Affiliations:** 1 Key Laboratory of Beibu Gulf Offshore Engineering Equipment and Technology, Beibu Gulf University, Qinzhou 535011, China; dengjunlin@bbgu.edu.cn (J.D.); houruihan529@163.com (R.H.); 2 School of Computer, Electronics and Information, Guangxi University, Nanning 530004, China; 2113391006@st.gxu.edu.cn (Y.D.); 2213393029@st.gxu.edu.cn (Y.L.)

**Keywords:** text-to-speech, speech synthesis, diffusion probabilistic model, MixGAN, mel-spectrogram

## Abstract

Denoising diffusion probabilistic models (DDPMs) have proven to be useful in text-to-speech (TTS) tasks; however, it has been a challenge for traditional diffusion models to carry out real-time processing because of the need for hundreds of sampling steps during the iteration. In this work, a two-stage fast inference and efficient diffusion-based acoustic model of TTS, the Cascaded MixGAN-TTS (CMG-TTS), is proposed to address this problem. An active shallow diffusion mechanism is adopted to divide the CMG-TTS training process into two stages. Specifically, a basic acoustic model in the first stage is trained to provide valuable a priori knowledge for the second stage, and for the underlying acoustic modeling, a mixture combination mechanism-based linguistic encoder is introduced to work with pitch and energy predictors. In the following stage of processing, a post-net is used to optimize the mel-spectrogram reconstruction performance. The CMG-TTS is evaluated on datasets such as the AISHELL3 and LJSpeech, and the experiments show that the CMG-TTS achieves satisfactory results in both subjective and objective evaluation metrics with only one denoising step. Compared to other TTS models based on diffusion modeling, the CMG-TTS obtains a leading score in the real time factor (RTF), and both stages of the CMG-TTS are effective in the ablation studies.

## 1. Introduction

Deep learning-based speech synthesis techniques have played an increasingly important role in generating near-human voices, giving rise to many excellent models. Text-to-speech (TTS) synthesizes high-quality audio signals based on the input text sequences, with three models such as the text analysis frontend, the acoustic model, and the neural vocoder [1,2,3,4]. Before model training begins, the text sequences are passed through a text frontend, which is regularized and converted to phoneme sequences. The acoustic model converts the phoneme sequences into acoustic features of a time-domain spectrogram (e.g., mel-spectrogram). The neural vocoder converts the acoustic features into waveform features. A common training method is used to train the acoustic model and neural vocoder separately, and to synthesize the speech signal by passing the mel-spectrogram as an intermediate representation feature into the vocoder [5,6,7].

Neural networks based autoregressive models have demonstrated strong performance in TTS tasks, which use frame-by-frame prediction to summarize the predicted results of all frames to obtain the audio samples of the input text [8,9,10,11,12]. However, autoregressive models usually encounter problems such as word jumps, repetition, and slow inference, and non-autoregressive models have been the focus in order to address these challenges [13,14]. Non-autoregressive models usually use a forward feedback block structure to process the input phoneme sequences in parallel, which helps to increase the speed of synthesized audio. However, this parallel processing requires strict alignment between the text sequences and mel-spectrogram, which can be realized by using an external alignment tool such as the Montreal forced alignment (MFA), or a knowledge distillation approach [13,14]. Normalized flow and dynamic programming approaches can also be applied to the matching of monotonic alignment information between text sequences and a mel-spectrogram [15,16,17].

Denoising diffusion probabilistic models (DDPMs), as a powerful generative model, have recently been demonstrated to show strong modeling performance in image generation and speech synthesis [18,19,20,21]. The DDPMs are usually divided into two processing stages, which include diffusion and denoising, respectively. In the diffusion process, small random noise is combined with the data based on a *T*-step Markov chain, while in the denoising process the synthesized noise is gradually removed through a parameterized *T*-step Markov chain. The DiffSinger [22] was the first acoustic model to introduce the diffusion model to the application of music generation, in which the noise signal was converted into a mel-spectrogram conditioned by the music score. In order to further improve speech quality and speed up the inference, a shallow diffusion mechanism is used in the DiffSinger to make full use of the prior knowledge extracted from the basic model, and the boundary prediction method is trained to determine the denoising step *T*. In this regard, the number of denoising steps can be reduced to 70. In a following effort, the DiffGAN-TTS [23] adopted generative adversarial network (GAN) to model the denoising distribution, and an active shallow diffusion structure similar to the DiffSinger was applied to further reduce the denoising step to 1. It can be seen that diffusion-based speech synthesis models are able to achieve stable and efficient training by optimizing the evidence lower bound (ELBO).

In this research, we concentrate on the acoustic modeling of speech, employing a two-stage architecture named Cascaded MixGAN-TTS (CMG-TTS) to optimize the performance of synthesized audio and the efficiency of model inference. The CMG-TTS incorporates a linguistic encoder featuring a mixture alignment structure. Additionally, pitch and energy predictors were integrated into this encoder, which significantly contributed to enhancing the prosody of the output audio. To improve the reconstructability of the mel-spectrogram, a post-net mechanism grounded in a deep convolutional network was implemented. Regarding the denoising process, the GAN structure was adopted. This approach effectively circumvents the need for the numerous denoising steps that are typical in diffusion models when using Gaussian functions. Moreover, an active shallow diffusion mechanism was applied to further curtail the required number of denoising steps. The CMG-TTS was evaluated on the AISHELL3 [24] and LJSpeech datasets, respectively. The experimental results demonstrate that even with only a single denoising step, satisfactory outcomes can be achieved in terms of the predicted mel-spectrogram, attention alignment, and audio quality. These findings not only validate the effectiveness of the proposed CMG-TTS architecture but also provide valuable insights for future research in the field of speech synthesis.

The rest of the paper is organized as follows: Section 2 shows the general structure of diffusion models, and Section 3 proposes the theory of the CMG-TTS model. Section 4 demonstrates the training and testing processes of the CMG-TTS, and Section 5 gives a discussion of the results. Finally, the conclusions are given in Section 6.

## 2. Diffusion Model

**Figure 1:**
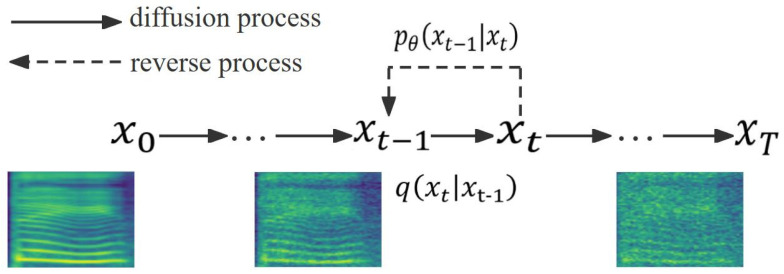
The directed graph for diffusion model.

The diffusion model includes a diffusion step followed by a denoising process. The diffusion process is a forward process, in which the data are influenced by small random noise until the data are completely distorted after *T* steps. The denoising process is an inverse process, and the polluted data are then recovered by learning a denoising function to gradually remove the added noise. Diffusion models usually require thousands of denoising steps to achieve the expected results, and the detailed process of the diffusion model is shown in Figure 1.

**Diffusion process:** In the diffusion step, a small amount of random Gaussian noise is added to the initial data $x_{0}$ step by step, and the resulting collapsed data $x_{T}$ are obtained. As shown in Equations (1) and (2), the diffusion process is based on a given predefined variance schedule $\beta_{t}$, with independently distributed variance $\beta_{1:T}$ at each step.

(1) $$q\left(x_{1:T}|x_{0}\right)=\prod_{t\geq 1}q\left(x_{t}|x_{t-1}\right)$$

(2) $$q\left(x_{t}|x_{t-1}\right)=N\left(x_{t};\;\sqrt{1-\beta_{t}}x_{t-1},\;\beta_{t}I\right)$$

where $q\left(x_{1:T}|x_{0}\right)$ denotes the diffusion process from $x_{0}$ to $x_{T}$, and N represents the normal distribution.

**Denoising process:** In the denoising process, $x_{T-1:0}$ is denoised iteratively from $x_{T}$ to obtain the final recovered data $x_{0}$, which is modeled by the parameterized θ. Equations (3) and (4) express the denoising process, such that

(3) $$p_{\theta }\left(x_{0:T}\right)=p\left(x_{T}\right)\prod_{t\geq 1}p_{\theta }\left(x_{t-1}|x_{t}\right)$$

(4) $$p_{\theta }\left(x_{t-1}|x_{t}\right)=N\left(x_{t-1};\mu_{\theta }\left(x_{t},t\right),\sigma_{t}^{2}I\right)$$

where $p_{\theta }\left(x_{0:T}\right)$ denotes the stepwise elimination of Gaussian noise from the diffusion sample $x_{T}$. To obtain $x_{0}$, $\mu_{\theta }\left(x_{t},t\right)$ and $\sigma_{t}^{2}$ denote the mean and variance, respectively.

In general, a Gaussian function is used to model the denoising distribution $p_{\theta }\left(x_{t-1}|x_{t}\right)$. The diffusion model can be optimized through maximizing the likelihood of $p_{\theta }(x_{0})$ and the evidence lower bound (ELBO$\;\leq \;\log\;p_{\theta }(x_{0})$). Under the impetus of the ELBO, the diffusion model compels $q\left(x_{t-1}|x_{t}\right)$ to converge towards the actual denoising distribution $p_{\theta }\left(x_{t-1}|x_{t}\right)$. Mathematically, the ELBO is expressed as follows:

(5) $$\mathrm{ELBO}=\sum_{t\geq 1}E_{q\left(x_{t}\right)}\left[D_{\mathrm{KL}}\left(q\left(x_{t-1}{|x}_{t}\right)||p_{\theta }\left(x_{t-1}|x_{t}\right)\right)\right]+C$$

where $D_{KL}$ represents the Kullback–Leibler (KL) scatter, and C denotes the constant term that does not depend on θ.

## 3. The CMG-TTS Model

Based on the diffusion model of GAN, the number of denoising steps can be further reduced by using an active shallow diffusion mechanism. This section describes the design motivation and the components of the CMG-TTS model, as well as the training loss.

### 3.1. Motivation

Diffusion models show powerful modeling capabilities for signal processing; however, they suffer from difficulty in the real-time processing of iterative Gaussian application [20]. There have been a number of studies focused on the synthesis speed of diffusion models, and one of the effective approaches is to use a GAN instead of a Gaussian function for denoising distribution [21,23]. DiffGAN-TTS is a model of this kind that can achieve fast and high-quality synthesized audio. The active shallow diffusion mechanism of the DiffGAN-TTS method can be further considered by introducing a two-stage cascaded training structure. The underlying acoustic model in the first stage of processing is designed to provide strong a priori knowledge for the diffusion model in the following stage. DiffGAN-TTS uses the classic FastSpeech2 for the underlying acoustic model, which suffers from hard alignment between the phoneme and mel-spectrogram.

**Figure 2:**
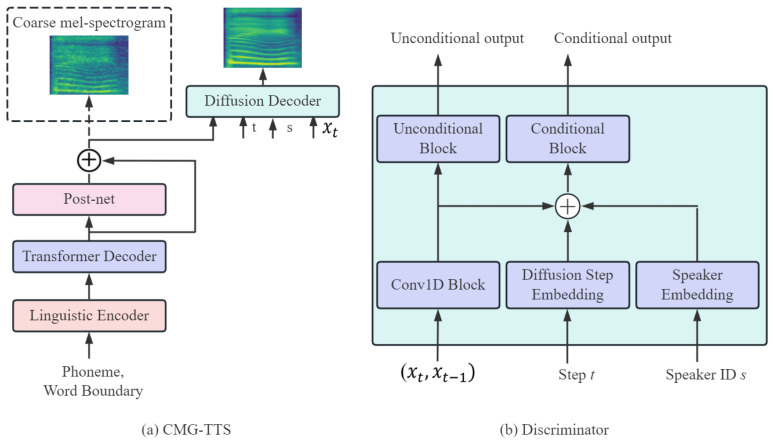
The overall architecture for the CMG-TTS scheme. The dashed box indicates the training stage of the basic acoustic model.

In this work, we introduce the DiffGAN-TTS with an active shallow diffusion structure to optimize the quality of the synthesized audio, and its training process can be explained with two stages. The underlying acoustic model in the first stage provides strong a priori knowledge for the diffusion model in the second stage. In terms of the basic acoustic model, we consider a linguistic encoder with pitch and energy predictors based on a mixture of alignment mechanisms, employing soft alignment of phonemes and hard alignment at the word level. Meanwhile, the mel-spectrogram reconstruction capability is investigated by introducing a post-net convolution network. Figure 2 shows the structure of the CMG-TTS scheme. It can be seen that the CMG-TTS contains a linguistic encoder, a transformer decoder, a post-net, a discriminator, and a diffusion decoder. In the first stage, the basic acoustic model in the CMG-TTS is trained to generate a coarse mel-spectrogram, as shown in the dashed box in Figure 2a, and then the coarse mel-spectrogram is fed to the input of the diffusion decoder to provide strong prior knowledge for training the denoising model in the second stage; in this way, the number of denoising steps T can be further reduced.

### 3.2. The Basic Model Architecture

**Figure 3:**
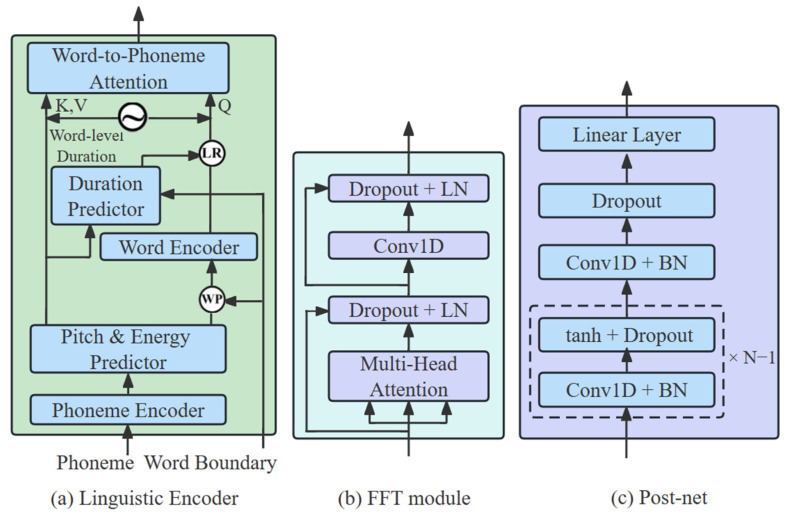
The basic acoustic structure of the CMG-TTS.

In this work, we introduce an active shallow diffusion mechanism that divides the model training into two stages. Specifically, we first train a basic acoustic model to provide strong a priori knowledge for training the denoising model in the second stage, which further reduces the number of denoising steps, T. Figure 3 shows the structure of the basic acoustic model of the CMG-TTS, which includes a linguistic encoder, a transformer decoder, and a post-net. The linguistic encoder processes the phoneme sequences into phoneme hidden sequences, and then the decoder converts the phoneme hidden sequences into a mel-spectrogram. The post-net further optimizes the mel-spectrogram and enhances the reconstruction capability.

**Linguistic Encoder and Transformer Decoder:** Figure 3a shows the structure of the linguistic encoder, in which “LR” denotes the length regulator, “WP” is the word-level pooling, and the sinusoidal-like symbol denotes relative position encoding [25]. The linguistic encoder contains the components of phoneme encoder, pitch predictor, energy predictor, word encoder, duration predictor, and word-phoneme attention model. The phoneme encoder, word encoder, and transformer decoder share a similar model structure based on a forward feedback transformer (FFT). Figure 3b shows the structure of the FFT, which contains multi-head self-attention, Dropout, and linear normalization (LN). It is obvious that a similar structure is applied to all the pitch, energy, and duration predictors, with a 2-layer 1D convolution network equipped with ReLU activation, linear normalization, and Dropout. The hidden states are projected into the output sequence with an extra linear layer.

**Post-net:** Figure 3c depicts a schematic diagram of the post-net architecture, which consists of a 5-layer 1D convolutional network. Each layer incorporates 512 units of 5 × 1 convolutional kernels. Subsequent to the convolution operation in each layer, batch normalization (BN) is applied to standardize the input features across the mini-batch, effectively accelerating the training process and reducing the risk of overfitting. This is followed by a dropout operation, which randomly “drops out” a fraction of the input units, further enhancing the model’s generalization ability. The Tanh activation function is then employed to introduce non-linearity into the network, enabling it to learn complex patterns in the data. Finally, the mel-spectrogram output is derived from the last layer through a linear mapping operation. This linear transformation projects the high-dimensional feature representation learned by the post-net onto the appropriate mel-spectrogram space.

### 3.3. Diffusion Decoder and Discriminator

**Figure 4:**
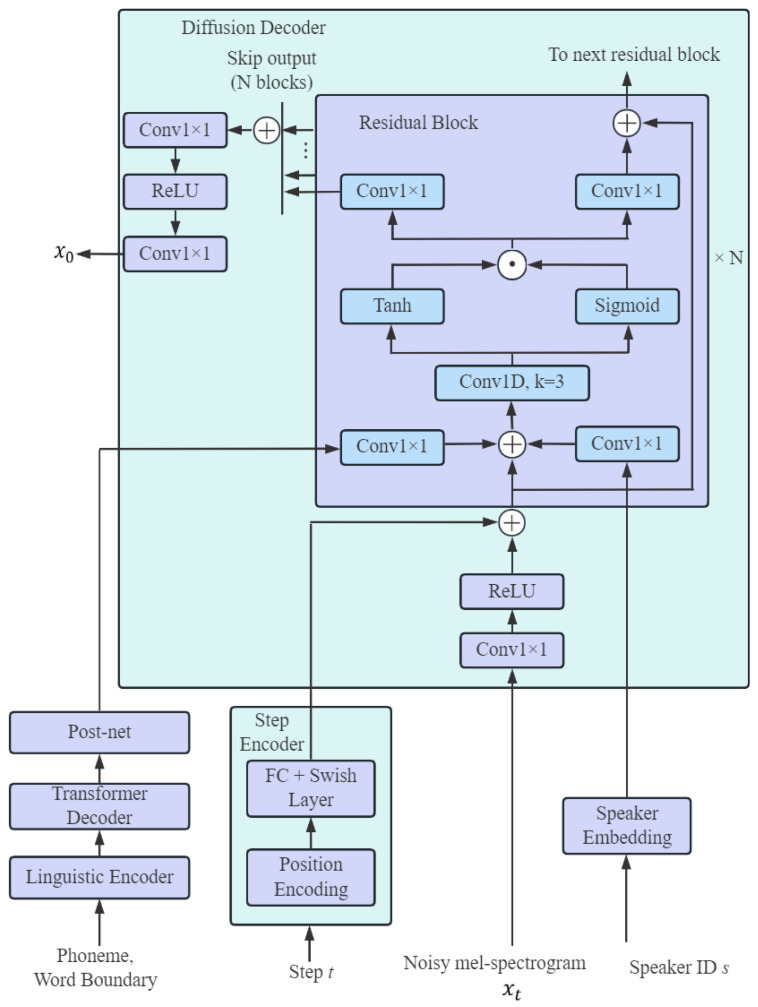
The architecture for diffusion decoder.

In the CMG-TTS, a diffusion decoder is designed for the denoising distribution using conditional GAN. The speed of model synthesis is accelerated by increasing the denoising steps size and reducing the denoising processes. Figure 4 demonstrates the structure of the diffusion decoder. The underlying structure contains 20 non-causal residual blocks with hidden states of 256. The diffusion decoder receives the output of the basic acoustic model, and the diffusion process encodes t, the noise mel-spectrogram $x_{T}$, and the speaker embedding s. The final output from the diffusion decoder can be calculated by sequentially examining the residual block with the skip connection layer, alternating between the convolution network and ReLU function, respectively.

In each denoising step $D_{adv}$, a discriminator is applied to compute the convergence between the actual denoising distribution $q\left(x_{t-1}|x_{t}\right)$ and the predicted denoising distribution $p_{\theta }\left(x_{t-1}|x_{t}\right)$, which is trained by the least-squares GAN (LS-GAN) loss [26]. In the joint conditional and unconditional loss (JCU Loss) [27], the accuracy of the mel-spectrogram and waveform mapping can be further improved. The discriminator is a CNN structure and the Conv1D block contains a 3-layer 1D convolution network and a LeakyReLU activation function, as shown in Figure 2b. The conditional and unconditional blocks share the same structure and consist of a 2-layer 1D convolution network. The discriminator can be modeled by $D_{\varphi }\left(x_{t-1},x_{t},t,s\right)$, taking the input of the data with noise $x_{t}$, the predicted mel-spectrogram $x_{t-1}$, the diffusion step with t, and the speaker with s.

### 3.4. Active Shallow Diffusion Mechanism

The loss function of many speech synthesis models is based on a single mean square error (MSE) or mean absolute error (MAE); however, there have been over-smoothing problems due to incorrect uni-modal distribution hypothesis of data [23]. In order to improve the performance of model synthesis, an active shallow diffusion mechanism is introduced in the CMG-TTS, in which the basis acoustic model with parameter φ is defined as $G_{\varphi }^{basic}\left(y,s\right)$ and trained using the minimization scheme, as follows:

(6) $$\min\;\sum_{t\geq 0}E_{q\left(x_{t}\right)}\left[Div\left(q_{diff}^{t}\left(G_{\varphi }^{basic}\left(y,s\right)\right),q_{diff}^{t}\left(x_{0}\right)\right)\right]$$

where $Div\left(\cdot \right)$ measures the scatter between the predicted and the actual values. $q_{diff}^{t}\left(\cdot \right)$ denotes the diffusion sampling function at step t, $E_{q\left(x_{t}\right)}$ represents the expectation with respect to the diffused samples, and $x_{t}=q_{diff}^{t}\left(x_{0}\right)$ means that $x_{0}$ is applied with the diffusion sampling function at step t to obtain the diffusion sample $x_{t}$.

**Figure 5:**
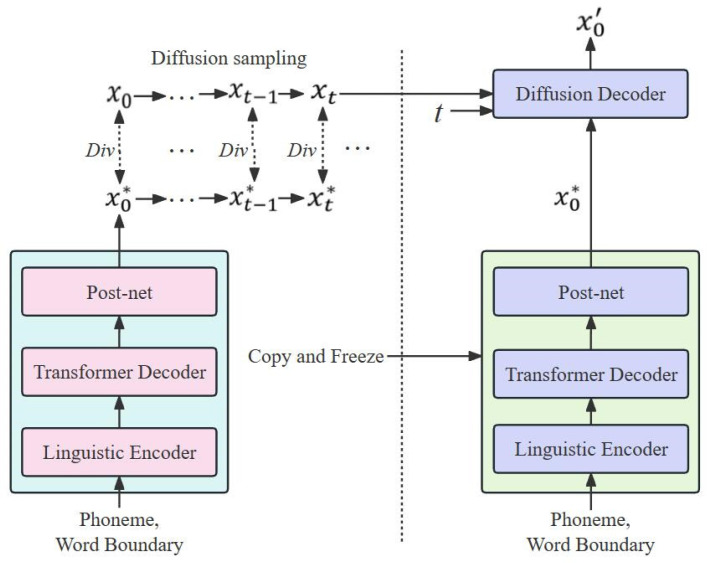
The two-stage cascaded training scheme.

Figure 5 shows the two-stage cascaded training scheme. In this structure, the noise samples generated by the basic acoustic model are trained to be as close to the diffuse samples in the real data as possible by continuously reducing the scatter between these samples. In the second stage, the pre-training weights of the basic acoustic model are first replicated to initialize the corresponding weights following a freezing operation. The diffusion decoder then receives the coarse mel-spectrogram $x_{0}^{*}$ and continues the training of the diffusion sampling and the denoising process. In the inference stage, the basic acoustic model produces the coarse mel-spectrogram $x_{0}^{*}$, and the diffusion sample $x_{1}^{*}$ is obtained through a diffusion step. The diffusion decoder takes the diffusion sample $x_{1}^{*}$ as a priori knowledge and obtains the final output $x_{0}^{\prime }$ through a denoising step.

### 3.5. Training Loss

The CMG-TTS is trained based on the generator loss as well as discriminator loss. The generator loss consists of the feature matching loss of $L_{fm}$ [28], acoustic reconstruction loss $L_{recon}$, and denoising convergence loss $L_{adv}$, as defined in Equations (7)–(9), respectively.

(7) $$L_{fm}=E_{q\left(x_{t}\right)}\left[\sum_{i=1}^{N}||D_{\varphi }^{i}\left(x_{t-1},x_{t},t,s\right)-D_{\varphi }^{i}\left(x_{t-1}^{'},x_{t},t,s\right)|{|}_{1}\right]$$

(8) $$L_{recon}=L_{mel}+L_{postnet}+\lambda_{d}L_{duration}+\lambda_{p}L_{pitch}+\lambda_{e}L_{energy}+L_{helper}$$

(9) $$L_{adv}=\sum_{t\geq 1}E_{q\left(x_{t}\right)}E_{p_{\theta }\left(x_{t-1},x_{t}\right)}\left[{\left(D_{\varphi }\left(x_{t-1},x_{t},t,s\right)-1\right)}^{2}\right]$$

where *N* denotes the number of hidden layers in the discriminator. $\lambda_{d}$, $\lambda_{p}$, and $\lambda_{e}$ denote the setting loss weights, and they are all set to 0.1. $L_{mel}$ and $L_{postnet}$ are based on the MAE loss, $L_{duration}$, $L_{pitch}$ and $L_{energy}$ are based on the MSE loss, and $L_{helper}$ is based on the Guided Attention Loss [29]. $L_{fm}$ is a similarity metric in the discriminator that distinguishes the real and generated data, and is obtained by accumulating the $l_{1}$ distance between them. $L_{recon}$ represents the basis reconstruction loss, and $L_{adv}$ indicates the convergence between the actual denoising distribution $q\left(x_{t-1}|x_{t}\right)$ and the denoising model distribution $p_{\theta }\left(x_{t-1}|x_{t}\right)$. The generator is trained by minimizing $L_{G}$, such that

(10) $$\;L_{G}=L_{fm}+L_{recon}+L_{adv}$$

In the second stage of training, the basis reconstruction loss $L_{recon}$ is set to 0, except for $L_{mel}$, in order to highlight the contribution of the diffusion model. The discriminator is optimized by minimizing $L_{D}$, such that

(11) $$\;L_{D}=\sum_{t\geq 1}E_{q\left(x_{t}\right)q\left(x_{t-1}|x_{t}\right)}\left[{\left(D_{\varphi }\left(x_{t-1},x_{t},t,s\right)-1\right)}^{2}\right]+E_{p_{\theta }\left(x_{t-1}|x_{t}\right)}\left[D_{\varphi }{\left(x_{t-1},x_{t},t,s\right)}^{2}\right]$$

## 4. Experiments

In this section, the model configuration and the datasets for testing are introduced to verify the validity of the proposed model.

### 4.1. Experimental Setup

**Datasets:** The performance of the CMG-TTS model was comprehensively evaluated on two benchmark datasets, namely AISHELL3 and LJSpeech. The AISHELL3 dataset encompasses a total of 88,035 audio segments, contributed by 218 native Mandarin speakers, with an aggregate duration of 85 h. This dataset represents a diverse range of Mandarin speech characteristics, making it suitable for evaluating the model’s performance in tonal languages. On the other hand, the LJSpeech dataset contains 13,100 English audio clips, all originating from a single speaker, with an approximate total duration of 24 h. The LJSpeech dataset is widely used for evaluating the performance of speech-related models in the context of non-tonal languages, especially for English-language tasks. For the purpose of model training and evaluation, a stratified random sampling approach was employed. Specifically, 87,011 samples were randomly selected from the AISHELL3 dataset, and 12,076 samples were randomly chosen from the LJSpeech dataset to form the training set. This sampling strategy ensured that the training set was representative of the overall data distribution in each dataset. For validation and testing, 512 samples were randomly selected from each of the two datasets for both the validation set and the test set. This balanced sampling across datasets enabled a fair and accurate assessment of the model’s generalization ability. To prepare the data for model training, text sequences were first transformed into phoneme sequences. This was achieved using well-established libraries such as pypinyin for Mandarin Chinese, which accurately converted Chinese characters into their corresponding pinyin phonetic notations, and g2p_en for English, which mapped English graphemes to phonemes. Subsequently, mel-spectrograms were generated from the original audio waveforms. The sampling rate was set to 22,050 Hz, which is a commonly used rate in speech processing tasks, ensuring sufficient frequency resolution. With a frame length of 1024 samples, a hop length of 256 samples, and a frequency bin size of 80, the mel-spectrograms were calculated. This configuration effectively captured the short-term spectral characteristics of the audio signals, providing a suitable input representation for the CMG-TTS model.

**The training setup:** The CMG-TTS model was trained on a single NVIDIA 3060 GPU, with a batch size of 16 on both the AISHELL3 and LJSpeech datasets. A gradually decaying learning rate was used to train the CMG-TTS, with the initial learning rates set to $10^{-3}$ and $2\times 10^{-3}$ for the generator and discriminator, respectively. An Adam optimizer was applied to training the two-stage cascaded scheme with $\beta_{1}$ = 0.9, $\beta_{2}$ = 0.98, and ϵ = $10^{-9}$ in the first train stage and $\beta_{1}$ = 0.5, $\beta_{2}$ = 0.9, and ϵ = $10^{-9}$ in the second stage. For the AISHELL3 dataset, the basic acoustic model in the first stage reached convergence after 360 k steps, and the diffusion model in the second stage reached convergence after 800 k steps. For the LJSpeech dataset, the first and second stages were trained for 300 k and 700 k steps, respectively, before convergence was reached. During our experiments, all models were used to obtain the final audio samples using the HiFi-GAN vocoder. This experiment was carried out on a CUDA version of 11.6 with Python 3.8 and Pytorch 1.8.0+cu111.

**The evaluation method:** The model performance could be measured using mean opinion score (MOS) [30], with the scores ranging from 1 to 5 with a 95 percent confidence interval. The quality of the modeled synthesized audio was further measured with objective evaluation metrics methods such as the mel-cepstral distortion (MCD) [31], F_0_ root mean squared error (F_0_ RMSE), perceptual evaluation of speech quality (PESQ) [32], short-time objective intelligibility (STOI) [33], segment signal-to-noise ratio (SegSNR), and real time factor (RTF). The RTF shows the speed of inference on a single GPU, giving the time needed for one second of audio. During the MOS, MCD, and F_0_ RMSE evaluations, the sample rate of the audio was set to 22,050 Hz. For the PESQ, STOI, and SegSNR, the sampling rate was set to 16,000 Hz to satisfy the computation needs.

### 4.2. Experimental Results

The tests were performed on the FastSpeech2, PortaSpeech, DiffSpeech (*T* = 64), DiffGAN-TTS (*T* = 4), and DiffGAN-TTS (two-stage) to compare with the proposed scheme; all these models were trained and reasoned on the basis of publicly available code, and these hyperparameters were all tuned by the original author. DiffSpeech denotes the DiffSinger applied to the TTS domain using the shallow diffusion mechanism. In the experiments, the number of denoising steps for DiffSpeech was set to 64. DiffGAN-TTS (*T* = 4) indicated that the training process was carried out using four steps of denoising, and the DiffGAN-TTS represented the use of a two-stage training scheme. The results of the experiments on the AISHELL3 and LJSpeech datasets are shown in Table 1 and Table 2.

**Audio Quality:** The experimental results on both datasets show that the CMG-TTS achieved the highest audio quality with only one denoising step. The CMG-TTS obtained MOS scores of 4.03 and 4.08, respectively, outperforming other TTS models in the tests. Meanwhile, the CMG-TTS achieved satisfactory performance in the objective metrics of MCD, F_0_ RMSE, PESQ, STOI, and SegSNR.

**RTF:** The CMG-TTS showed an efficient sampling capability to synthesize a high-fidelity mel-spectrogram in only one denoising step. The RTF value of the different TTS models was evaluated on the two datasets, and it can be seen that the CMG-TTS effectively reduced the inference time compared to other diffusion-based TTS models.

**Figure 6:**
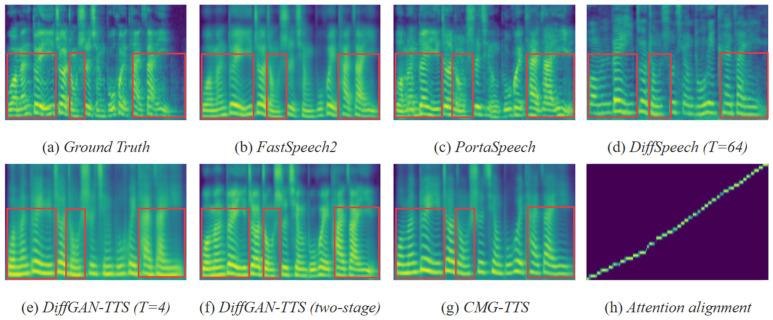
Visualization of the mel-spectrogram for different TTS models.

**Visualizations:** The mel-spectrograms of the CMG-TTS and other TTS models were compared on the AISHELL3 dataset under the same text sequence conditions as shown in Figure 6. The input phoneme sequence was “w uo3 m en5 d uei4 y i2 zh e4 zh ong3 sh iii4 q ing5 b u2 h uei4 t ai4 z ai4 y i4”. The CMG-TTS exhibited competitive performance in the low- and medium-frequency regions, while maintaining high quality in the high-frequency regions. The attention convergence graph during the training is presented in Figure 6h, and it can be seen that the attention alignment was clear and smooth, which indicates that the CMG-TTS achieved satisfactory performance in aligning the text sequences with the mel-spectrogram.

### 4.3. Ablation Tests

Ablation tests also had to be performed on the CMG-TTS to validate the effectiveness of the individual structures, including the post-net and active shallow diffusion mechanism. In the ablation test, the CMG-TTS was trained with only the active shallow diffusion model removed, which would however result in unmanageable errors. Table 3 shows the test results on the AISHELL3 dataset, and it demonstrates that the CMG-TTS elimination of the post-net resulted in a significant degradation of the audio quality. Meanwhile, it is obvious that there was a small reduction in the audio quality of the CMG-TTS after eliminating both the post-net and the active shallow diffusion structure. Our ablation studies validate the CMG-TTS structure.

## 5. Discussion

In previous studies, end-to-end speech synthesis models have been an important task in the field of TTS, and fully end-to-end models have been the focus. For example, FastSpeech2s [14] adopts an auxiliary mel-spectrogram decoder and adversarial training to learn text representations, and a special spectral loss was used to mitigate the length mismatch between the target and generated speech. In a different effort, EATS [34] uses adversarial training and differentiable alignment methods while employing a soft dynamic time warping loss computed by dynamic programming to mitigate the length mismatch that exists between the generated speech and the target speech. Another model named EFTS-Wav [35] also used an auxiliary mel-spectrogram decoder for alignment learning. On this basis, the EFTS-Wav proposes an index mapping vector in the monotone alignment model, which led to the design of a novel monotone alignment mechanism. In a parallel effort, the Wave-tacotron [36] combines Tacotron2 and normalized flow to simplify maximizing the likelihood of the training data, and VITS [37] learns text alignment during training that maximizes the likelihood of data while improving expressiveness by leveraging variational reasoning and normalization flow in an adversarial structure. JETS [38] unites the FastSpeech2 and HiFi-GAN vocoder, and it designs a novel token duration that does not require an additional external speech-to-text alignment model. In this effort, the proposed CMG-TTS achieved better results in the evaluations; however, from the structure of the CMG-TTS, it can be seen that it cannot be considered as an end-to-end model, since a vocoder was still required to synthesize the final audio sample. Therefore, the CMG-TTS must be trained separately from the vocoder, which introduces additional training costs and error accumulation. In contrast, end-to-end models can generate audio in parallel in a short period of time and can reduce the error accumulation without extra time. Therefore, it is still necessary to study the scheme of a fully end-to-end training mechanism that does not affect the performance of CMG-TTS.

## 6. Conclusions

In this work, a two-stage processing scheme, the CMG-TTS, was introduced as an efficient speech synthesis model with an active shallow diffusion mechanism. In the first stage of processing, the CMG-TTS was trained to learn diffusion samples from real audio samples by training the underlying acoustic model, in which a linguistic encoder with a mixture alignment structure was used as the pitch and energy predictors. Then, a post-net with a 5-layer 1D convolution network was trained to optimize the reconstruction performance of the mel-spectrogram. In the second stage, a diffusion decoder was adopted to reduce the noise of the coarse samples and to obtain a high-fidelity mel-spectrogram. Finally, a HiFi-GAN vocoder was applied to convert the mel-spectrogram to the final audio output. The performance of the proposed CMG-TTS was evaluated on both the AISHELL3 and LJSpeech datasets. Specifically, a subjective assessment metric like MOS was used for the performance evaluation, in addition to objective assessment metrics such as the MCD, F0 RMSE, PESQ, STOI and SegSNR. The experimental results show that the CMG-TTS is able to synthesize high-fidelity audio samples with only one denoising step. Ablation tests were also carried out to assess the performance of the CMG-TTS, and it was proved that each of the structures in the CMG-TTS were effective.

## Figures and Tables

**Table 1:** Experimental results on the AISHELL3 dataset.

Method	MOS (↑)	MCD (↓)	F_0_ RMSE (↓)	PESQ (↑)	STOI (↑)	SegSNR (↑)	RTF (↓)
Ground Truth	4.27 ± 0.06	-	-	-	-	-	-
FastSpeech2	3.83 ± 0.07	17.808	0.724	1.061	0.146	−8.271	0.096
PortaSpeech	**4.02 ± 0.07**	17.665	**0.719**	1.069	0.158	−8.121	0.115
DiffSpeech (*T* = 64)	**4.04 ± 0.06**	17.721	0.748	1.059	0.157	−8.191	0.184
DiffGAN-TTS (*T* = 4)	3.93 ± 0.06	17.746	0.745	1.058	0.161	−8.177	0.167
DiffGAN-TTS (two-stage)	3.89 ± 0.07	17.704	0.783	1.054	0.155	−8.124	0.144
CMG-TTS	**4.03 ± 0.07**	**17.611**	0.743	**1.078**	**0.165**	**−** **7.858**	0.121

**Table 2:** Experimental results on the LJSpeech dataset.

Method	MOS (↑)	MCD (↓)	F_0_ RMSE (↓)	PESQ (↑)	STOI (↑)	SegSNR (↑)	RTF (↓)
Ground Truth	4.34 ± 0.07	-	-	-	-	-	-
FastSpeech2	3.94 ± 0.06	6.973	0.306	1.062	0.251	−6.049	0.044
PortaSpeech	4.06 ± 0.07	6.694	0.301	**1.074**	0.274	−5.781	0.071
DiffSpeech (*T* = 64)	**4.09 ± 0.05**	6.758	0.303	1.071	0.271	−5.793	0.126
DiffGAN-TTS (*T* = 4)	4.02 ± 0.06	6.801	0.309	1.599	0.268	−5.867	0.108
DiffGAN-TTS (two-stage)	3.99 ± 0.07	6.737	0.311	1.601	0.261	−5.691	0.096
CMG-TTS	**4.08** ± 0.06	**6.671**	**0.298**	1.667	**0.276**	**−5.661**	0.087

**Table 3:** Ablation study results.

Method	MOS (↑)	MCD (↓)	F_0_ RMSE (↓)	PESQ (↑)	STOI (↑)	SegSNR (↑)
CMG-TTS	**4.03 ± 0.07**	**17.611**	**0.743**	**1.078**	0.165	**−** **7.858**
Remove post-net	3.95 ± 0.07	17.707	0.776	1.063	0.146	−8.136
Remove post-net and two-stage	3.99 ± 0.06	17.645	0.756	1.075	**0.168**	−8.091

## Data Availability

Data are available upon request.
